# Mechanistic insights into lipoprotein(a)-induced cardiomyocyte ferroptosis via ROS/p38/p53 signaling

**DOI:** 10.3389/fmed.2026.1858249

**Published:** 2026-07-13

**Authors:** Yujia Li, Xi Chen, Chuan He, Yukai Zhang, Tiechao Jiang

**Affiliations:** 1 Department of Cardiology, China-Japan Union Hospital of Jilin University, Changchun, Jilin, China; 2 Department of Neurosurgery, The Second Hospital of Jilin University, Changchun, Jilin, China

**Keywords:** ferroptosis, lipoprotein(a), myocardial injury, p38, p53

## Abstract

**Objective:**

Lipoprotein(a) [Lp(a)], a low-density lipoprotein-like molecule covalently linked to apolipoprotein (a), is a residual cardiovascular risk factor with established atherogenic and antifibrinolytic properties. However, its direct involvement in cardiomyocyte injury mechanisms remains unclear. This study aimed to investigate the effects of Lp(a) on cardiomyocytes.

**Methods:**

A combination of *in vitro* cell culture and *in vivo* small animal models were used for investigations.

**Results:**

Lp(a) induced ferroptosis through a redox-sensitive pathway via sequential p38 MAPK activation and p53-mediated transcriptional regulation. Exposure of AC16 human cardiomyocytes to Lp(a) triggered hallmark ferroptotic events, including intracellular Fe^2+^ accumulation, an increase in malondialdehyde (MDA) levels, and concurrent increases in p38 MAPK (p-p38) phosphorylation. Pharmacological blockade of p38 using SB203580 or siRNA-mediated p38 silencing significantly attenuated these ferroptotic markers, confirming the central role of p38 in sensitizing cardiomyocytes to ferroptosis. p38 activation drove the nuclear translocation of p53, with both pharmacological p53 inhibition (pifithrin-α) and genetic p53 knockdown effectively mitigating Lp(a)-induced lipid peroxidation and cell death. Furthermore, Lp(a) promoted an increase in intracellular reactive oxygen species (ROS) levels and initiated p38 phosphorylation, subsequently activating p53 to suppress SLC7A11 expression. These cellular findings were validated *in vivo* using Lp(a)-treated C57BL/6J mice, which recapitulated cardiac dysfunction, as indicated by characteristic ferroptotic markers: myocardial Fe^2+^/MDA elevation, glutathione/cysteine depletion, and p38–p53 axis activation.

**Conclusion:**

Lp(a) activates p38 by increasing intracellular ROS levels and promotes ferroptosis in cardiomyocytes via SLC7A11 inhibition, which depends on p53 activation.

## Graphical Abstract

**Figure:**
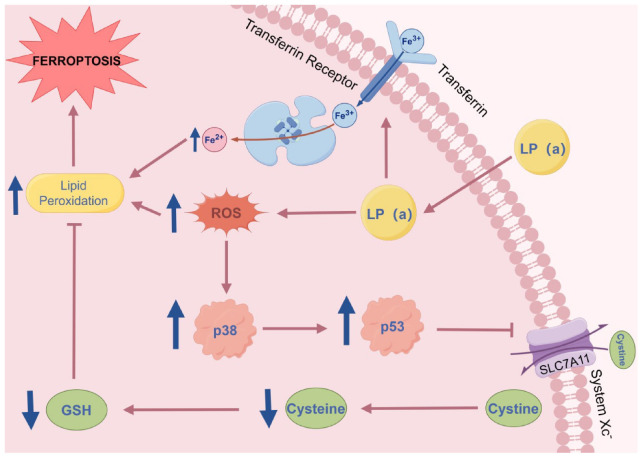
Schematic illustrating the role of p38 in Lp(a)-induced cardiomyocyte ferroptosis. Lp(a) upregulates the transferrin receptor 1, increases Fe^2+^ in cardiomyocytes, and upregulates intracellular reactive oxygen species (ROS) levels, leading to p38 activation. Activated p38 stimulates p53, which inhibits SLC7A11, ultimately promoting ferroptosis in cardiomyocytes via ROS/p38/p53 signaling.

## Introduction

Cardiovascular diseases are a leading cause of mortality worldwide (1), with elevated lipid levels recognized as a known risk factor. However, even after lowering LDL cholesterol levels, many patients remain at a high risk of cardiovascular disease events. Lipoprotein(a) [Lp(a)] is a lipoprotein similar to low-density lipoprotein (LDL) cholesterol, with a core consisting of LDL-like particles surrounded by an outer layer of covalently bound apolipoprotein(a). Elevated Lp(a) levels have been identified as a residual risk factor for cardiovascular events. A recent meta-analysis revealed an association of high Lp(a) levels with a higher risk of all-cause mortality and cardiovascular death in the general population and patients with cardiovascular disease (2). Furthermore, Lp(a) at concentrations > 93 mg/dL was associated with cardiovascular mortality in a Mendelian randomisation study (3). Given its core LDL particles, Lp(a) exhibits atherogenic, pro-inflammatory, and pro-coagulant effects and is associated with coronary atherosclerosis, aortic stenosis, aortic calcification, and atrial fibrillation. In previous studies focusing on the relationship between lipids and atherosclerosis, elevated Lp(a) levels were associated with increased incidences of myocardial fibrosis, myocardial scarring, and left atrial remodeling, indicating that Lp(a) may affect myocardial tissues (4). Moreover, Lp(a) can lead to heart failure, although some cases may be causally related to coronary atherosclerosis (5). However, the underlying mechanisms remain unclear.

Stress-responsive p38 mitogen-activated protein kinase (MAPK), a key member of the MAPK family, exerts its biological effects in response to phosphorylation (p-p38) to regulate downstream transcription factors and inflammatory mediators. Sustained activation of MAPK has been implicated in cardiovascular pathogenesis, including its dual role in promoting vascular smooth muscle cell apoptosis and osteogenic differentiation via calcification pathways (6). Mechanistically, MAPKK4 amplifies ischemia-reperfusion-induced coronary microvascular endothelial damage by phosphorylating p38, thereby potentiating apoptotic cascades in endothelial cells (7). Meanwhile, TGFβ/NOX4/reactive oxygen species (ROS)-driven p38 activation has been causally linked to myocardial fibrotic remodeling in experimental models (8). p38 activation has also been observed in animal models of heart failure, and myocardial biopsy samples obtained from patients with heart failure have revealed elevated p38 activity compared with that in “healthy” hearts (9–11). Collectively, these findings indicate an association between p38 and myocardial injury.

Ferroptosis is an iron-dependent form of regulated cell death driven by lipid peroxidation and triggered by disrupted redox homeostasis. It is characterized by glutathione (GSH) depletion, impaired GSH peroxidase 4 activity, and cystine/glutamate antiporter system Xc^–^ (xCT) dysfunction, resulting in a pathological accumulation of ferrous ions and ROS. Ferroptosis plays an important role in cardiovascular diseases through regulatory mechanisms mediated by the tumor suppressor p53. Under physiological conditions, p53 is constitutively degraded by murine double minute 2 and murine double minute 4. Meanwhile, in response to cellular stress-related stimuli, such as DNA damage, hypoxia, or oncogenic activation, post-translational modifications (phosphorylation and acetylation) stabilize p53, thereby initiating ferroptotic cascades. Mechanistically, p53 induces ferroptosis by transcriptionally repressing SLC7A11, the catalytic subunit of system Xc^–^, to block cystine import, thereby exacerbating GSH depletion and promoting ROS-driven oxidative stress (12). Concurrently, p53 activates spermidine/spermine N1-acetyltransferase 1 (SAT1), the rate-limiting enzyme in polyamine catabolism, which generates peroxidation-susceptible lipid species through polyamine oxidation, thereby amplifying lipid peroxidation and inducing ferroptosis (13). These coordinated events accordingly identify p53 as the central orchestrator of redox imbalance-mediated cell death in the cardiovascular system.

In the well-characterized p38 MAPK–p53 regulatory axis, activated p38 directly phosphorylates p53 at key residues (Ser15, Ser33, and Ser46) to enhance protein stability and transcriptional competence, whilst concurrently mobilizing downstream kinases, including ATF-2 and MNK1/2, to amplify p53-mediated signaling via synergistic post-translational modifications (14, 15). However, although the findings of previous studies using diabetic nephropathy and hepatocellular carcinoma models have established p38-driven p53 activation as a mediator of apoptosis and cell cycle arrest (16, 17), its roles in other cell death modalities have yet to be sufficiently determined. This study aimed to investigate the effects of Lp(a) and role of p38-driven p53 activation on cardiomyocytes.

## Materials and methods

### Lp(a) extraction

The study protocol was approved by the Ethics Committee of the Third Hospital of Jilin University, Changchun, China (approval number: 2024110703). Plasma was obtained by centrifuging blood collected from healthy volunteers. After adjusting the density with potassium bromide to 1.21, 1.1, 1.063, and 1.006 g/mL, Lp(a) was isolated using ultracentrifugation (densities layered in an ultrafiltration tube at volumes of 4.5, 3, 3, and 1 mL, and then centrifuged at 40,000 rpm/min at 10°C for 3 h), followed by purification with a HiTrap Q column (17115401, Cytiva, Shanghai, China). After determining the protein concentration of the Lp(a) sample by using the BCA kit (P0010, Beyotime Biotechnology, Shanghai, China), the Lp(a) concentration was measured using an Lp(a) ELISA kit (ab212165, Abcam, Shanghai, China). The comparison showed that the sample contained over 95% Lp(a). If the concentration was insufficient for experimental requirements, the sample was concentrated using a protein concentrator ultrafiltration device. The level of oxidized Lp(a) in the samples was detected using the ox-Lp(a) ELISA kit (ZY5126012, Zhuoyu Bio, Shanghai, China) to ensure that its content was qualified.

### Reagents and antibodies

SB203580, pifithrin-α, ferrostatin-1 (Fer-1), deferoxamine (DFO), ferric ammonium citrate (FAC), and *N*-acetylcysteine (NAC) were obtained from MedChemExpress (Shanghai, China), and H_2_O_2_ was provided by Sigma-Aldrich (Shanghai, China). The antibodies used in this study—GPX4 (30388-1-AP), ACSL4 (22401-1-AP), p38 (14064-1-AP), p-p38 (28796-1-AP), p53 (10442-1-AP), transferrin (TF) (17435-1-AP), transferrin receptor 1 (TFR1) (32381-1-AP), ferritin light chain (FT) (10727-1-AP), ferroportin (FPN) (26601-1-AP), β-actin (20536-1-AP), and SLC7A11 (26864-1-AP)—were all purchased from Proteintech (Wuhan, China).

### Cell culture

The AC16 human cardiomyocyte cell line, procured from American Type Culture Collection, was cultured in DMEM/F12 supplemented with 10% fetal bovine serum and 1% penicillin–streptomycin under standardized conditions (37 °C, 5% CO_2_, humidified atmosphere). Upon reaching 70–90% confluence, the cells were sub-cultured by detaching with trypsin-EDTA. Experiments were performed exclusively using cells in the logarithmic growth phase, characterized by optimal morphological integrity and viability.

### Cell survival/death assay

Cell survival was measured using the CCK-8 kit (C0038, Beyotime Biotechnology) and LDH assay kit (C0017, Beyotime Biotechnology) following the manufacturer’s instructions. Cardiomyocytes were treated with indicated interventions. After treatment, the supernatant of cell culture medium was collected and incubated with LDH working solution in dark conditions at room temperature. The absorbance value at 490 nm was detected by a microplate reader. Total cell lysis group was set as maximum LDH release control, and untreated normal cells served as background control. The cell death rate was calculated according to the formula provided by the kit manufacturer: Cell death rate (%) = (OD value of sample group he kit manufacturercalculated according d incubated lysis group sample group he kit ma × 100%.

### Small interfering RNA (siRNA) transfection

Lipofectamine 3000 (Thermo Fisher Scientific, Waltham, MA, United States) was used to transfect siRNA into cells following the manufacturer’s instructions. The siRNA sequences targeting p38 (5’-GGCACACAGAUGAUGAAAUTT-3’) and p53 (5’-CCCGGACGAUAUUGAACAAUG-3’), as well as scrambled siRNA (5’-UUCUCCGAACGUGUCACGUTT-3’), were obtained from GenePharma (Suzhou, China).

### Western blotting and co-immunoprecipitation

Western blotting and co-immunoprecipitation were performed as described previously (18).

### Measurement of Fe^2+^, malondialdehyde (MDA), GSH, cysteine, and ROS levels

Ferrous ion (S1066M), malondialdehyde (MDA; S0131M), GSH (S0052), cysteine (S0145M), and DCFH-DA (S0034M) assay kits were obtained from Beyotime Biotechnology and used according to the manufacturer’s instructions.

### Immunocytochemical staining

For immunocytochemical staining, the AC16 cells treated with Lp(a) were incubated with 1% Triton X-100 for 10 min. After the cells were fixed in ethanol and washed with PBS, the nonspecific binding sites were blocked with BSA. Then, the cells incubated overnight with the antibodies against p-p38 (1:100) or p53 (1:100), followed by incubation 1 h with goat anti-rabbit IgG (1:200) conjugated with Fluor 488 or Cy3 and counterstained 5 min with Heochst 33258. Eventually, the cells were observed by a fluorescence microscope.

### FerroOrange

AC16 cells were inoculated into culture dishes or 96-well plates and incubated overnight at 37°C in a 5% CO_2_ incubator. After replacing the medium containing the drug and leaving it to react for the indicated time, the culture medium was removed and the cells were washed three times with serum-free culture medium or PBS. The FerroOrange working solution (Dojindo Laboratories, Shanghai, China) was added at a concentration of 1 μmol/L, followed by incubation for 30 min at 37°C in a 5% CO_2_ incubator. The samples were used to measure the fluorescence intensity and observed under a fluorescence microscope.

### *In vivo* mouse models

Thirty-six male C57BL/6J mice (6 weeks old, 19–21 g) were obtained from Beijing Charles River Laboratories and housed in a pathogen-free environment under a 12-h light/dark cycle with free access to water and food. The study protocol was approved by the Ethics Committee of the First Hospital of Jilin University (approval number 20240426).

The mice were randomly assigned to one of the following six groups: Group 1, intravenously administered saline via the tail vein; Group 2, intraperitoneally administered SB203580 (5 mg/kg/day) and intravenously administered saline via the tail vein; Group 3, intraperitoneally administered pifithrin-α (2.2 mg/kg/day) and intravenously administered saline via the tail vein; Group 4, intravenously administered Lp(a) (15 mg/kg/day) via the tail vein; Group 5, intraperitoneally administered SB203580 (5 mg/kg/day) and intravenously administered Lp(a) (15 mg/kg/day) via the tail vein; and Group 6, intraperitoneally administered pifithrin-α (2.2 mg/kg/day) and intravenously administered Lp(a) (15 mg/kg/day) via the tail vein. Following three consecutive days of injections, cardiac function parameters were measured in each group of mice using small-animal ultrasound. Animals were euthanized by cervical dislocation, and heart tissues were harvested. The tissues were fixed in formalin and embedded in paraffin.

### Statistical analysis

The data analyzed in this study were based on the results obtained from more than three replicate treatments. Statistical analyses were performed using SPSS17.0 and GraphPad Prism 9 software. The data are expressed as the means ± standard deviation. *Post-hoc* power analysis for one-way ANOVA was performed using G*Power 3.1.9.2 based on our original experimental data. The calculated statistical power exceeded the commonly accepted cutoff value of 0.8, confirming that the current sample size was sufficient to detect statistically significant intergroup differences. All statistical outcomes were interpreted according to the preset significance threshold (*P* < 0.01). Consistent with the conventional reporting practice in cell biology, only significance levels were presented rather than exact *P-*values. In the *in vitro* cellular experiment, six parallel wells per group (*n* = 6) were designed as technical replicates using AC16 cells from the same batch. Independent biological repeats were conducted on separate days with freshly cultured cells to verify result reproducibility. Cells were randomly dispensed into different wells during seeding and drug treatment. Random grouping was also implemented for animals in *in vivo* experiments.

## Results

### Lp(a) induces ferroptosis in cardiomyocytes

**FIGURE 1:**
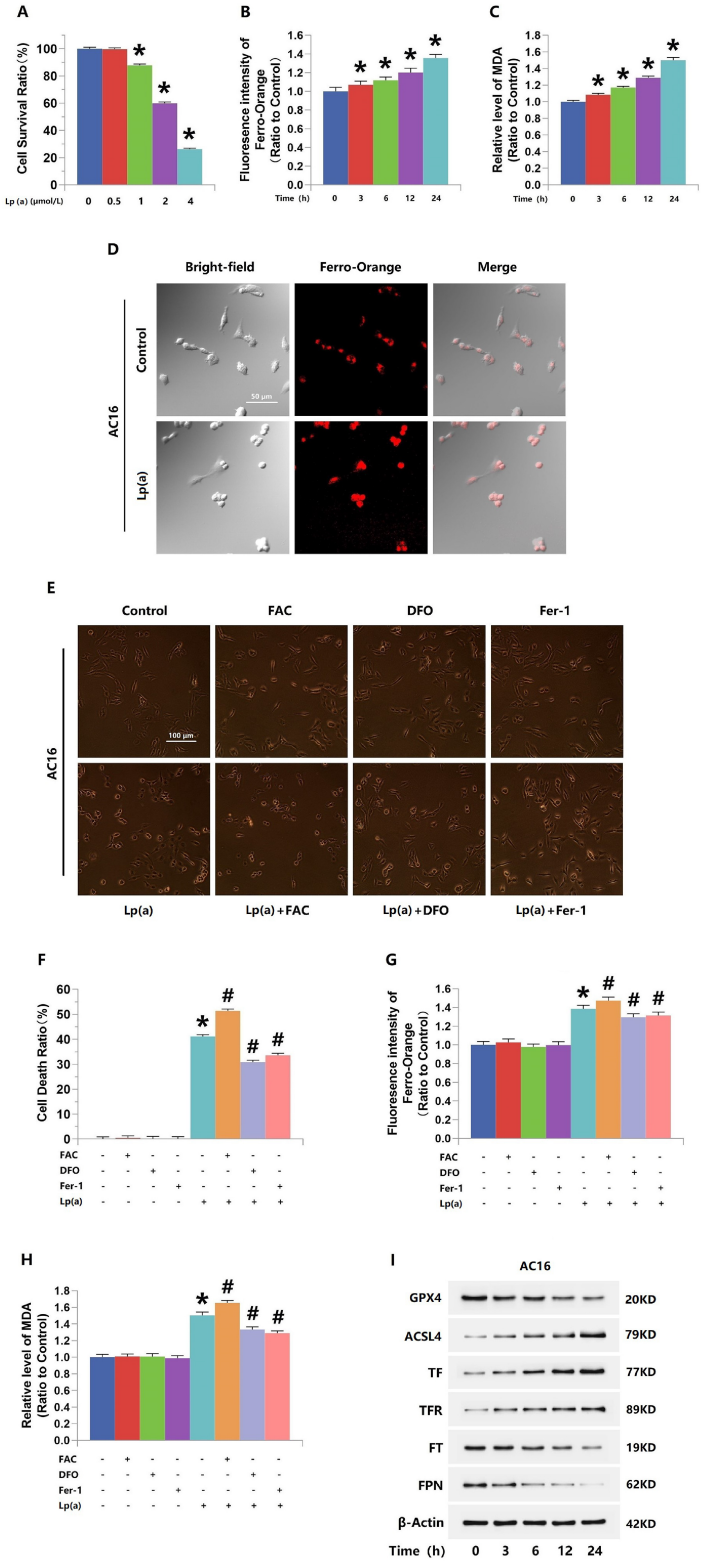
Lp(a) induces ferroptosis in cardiomyocytes. **(A)** Treatment of AC16 cells with Lp(a) at concentrations of 0, 1, 1.5, 2, and 4 μmoL/L for 24 h revealed a concentration-dependent cytotoxicity, with 2 μmol/L Lp(a) reducing cell viability to 50% compared with untreated controls. **(B)** Time-course analysis using 2 μmoL/L Lp(a) exposure demonstrated progressive ferroptotic changes. Specifically, FerroOrange fluorescence intensity significantly increased after a 3-h treatment, with maximal enhancement observed at 12 and 24 h. **(C)** Intracellular MDA levels, a lipid peroxidation marker, exhibited time-dependent elevation throughout the 2 μmoL/L Lp(a) exposure for 0, 3, 6, 12, and 24 h. **(D)** Confocal microscopy confirmed intensified FerroOrange fluorescence in 2 μmoL/L Lp(a)-treated cells compared with the controls after 24 h of exposure. **(E)** Optical microscopy analysis of AC16 cells pre-treated with 100 μmoL/L FAC, 100 μmoL/L DFO, or 20 μmoL/L Fer-1 for 1 h prior to 2 μmoL/L Lp(a) exposure for 24 h revealed distinct morphological alterations. **(F–H)** Pre-treatment with DFO and Fer-1 for 1 h prior to Lp(a) exposure substantially attenuated cell death, FerroOrange fluorescence, and MDA levels. However, pre-treatment with FAC for 1 h prior to Lp(a) exposure substantially aggravated cell death, FerroOrange fluorescence, and MDA levels. **(I)** Western blotting showed lipid peroxidation and dynamic regulation of iron metabolism proteins, with 2 μmoL/L Lp(a) treatment altering expression levels of GPX4, ACSL4, transferrin, transferrin receptor 1, ferroportin, and ferritin across 0–24 h exposure durations. *P < 0.01 versus the control group; ^#^*P* < 0.01 versus the Lp(a) group. The values are expressed as mean ± SD (*n* = 6 per group).

To investigate Lp(a)-induced ferroptosis in cardiomyocytes, AC16 cells were treated with Lp(a) at concentrations of 0, 1, 1.5, 2, and 4 μmoL/L for 24 h, which resulted in a 50% reduction in viability at 2 μmoL/L (Figure 1A). FerroOrange probing revealed time-dependent intracellular ferrous iron accumulation (Figure 1B), corroborated by elevated levels of the ferroptosis-associated lipid peroxidation product MDA (Figure 1C) and an intensification of FerroOrange fluorescence under a confocal microscope (Figure 1D). Supplementation with 100 μmoL/L FAC promoted increases in ferrous iron levels, MDA production, and cell death, whereas treatment with 100 μmoL/L the iron chelator DFO reversed these effects. Similarly, 20 μmoL/L Fer-1, a lipid peroxidation inhibitor, attenuated both MDA accumulation and cell death (Figures 1E–H), thereby confirming that lipid peroxidation is the primary driver of Lp(a)-mediated cytotoxicity. Western blotting for markers of lipid peroxidation and iron-regulatory proteins revealed time-dependent upregulation of ACSL4, TFR1, and TF alongside a concomitant downregulation of GPX4, FT, and FPN (Figure 1I), indicating enhanced cellular iron uptake, impaired iron storage and export, and resultant intracellular iron overload. Collectively, these findings suggest that Lp(a) perturbs cardiac iron homeostasis, exacerbates lipid peroxidation, and triggers ferroptosis in cardiomyocytes.

### p38 sensitizes cardiomyocytes to Lp(a)-induced ferroptosis

**FIGURE 2:**
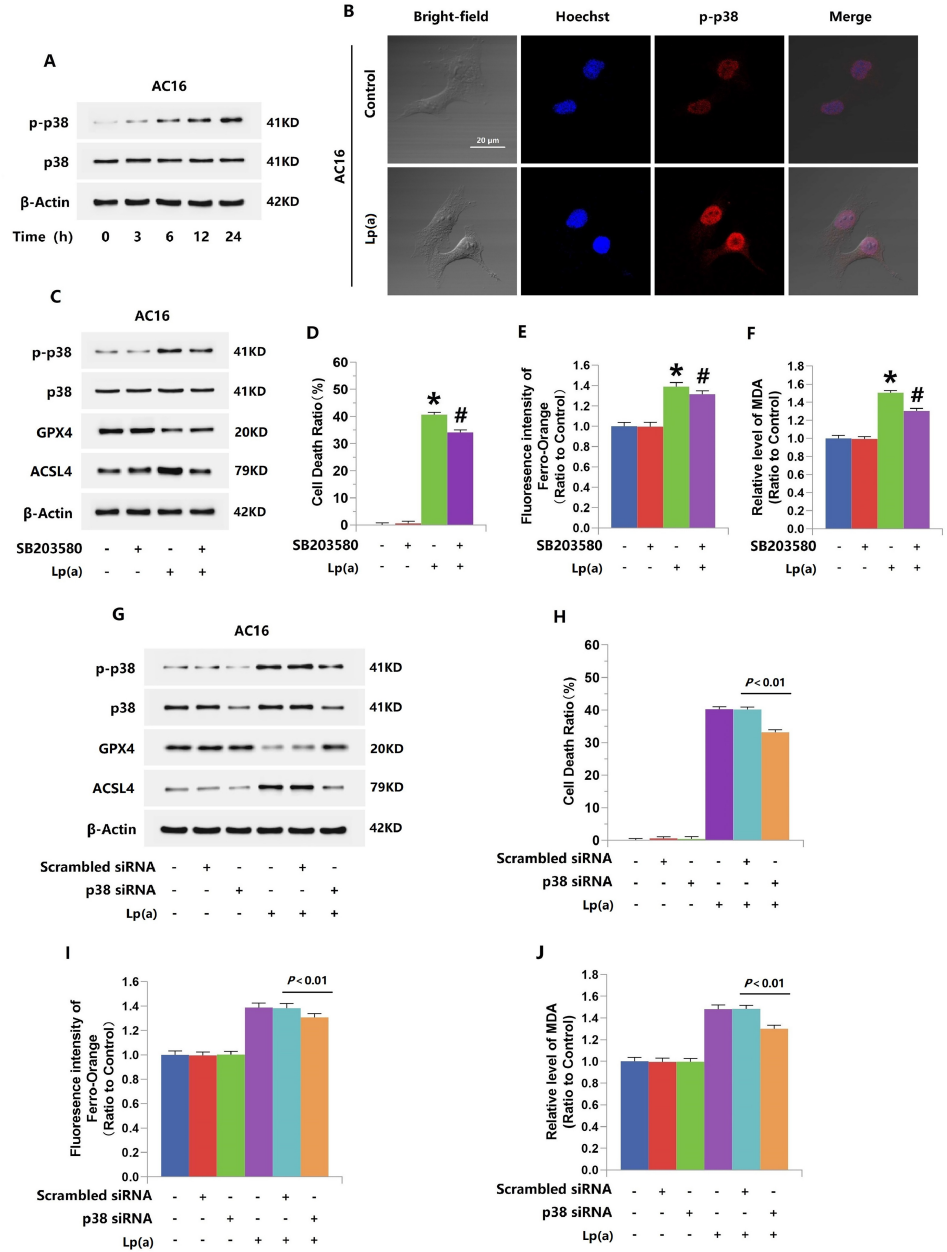
p38 sensitises cardiomyocytes to Lp(a)-induced ferroptosis. **(A)** Time-dependent upregulation of phosphorylated p38 (p-p38) protein levels was observed in AC16 cells treated with 2 μmol/L Lp(a) for 0, 3, 6, 12, and 24 h. **(B)** Confocal microscopy revealed increased p-p38 expression in both the cytoplasm and nuclei of AC16 cells following 24-h exposure to 2 μmol/L Lp(a). **(C)** Pre-treatment with 10 μmol/L SB203580 for 1 h attenuated the Lp(a)-induced upregulation of p-p38 and ACSL4 expression, as well as the downregulation of GPX4 expression. **(D)** SB203580 pre-treatment for 1 h suppressed Lp(a)-triggered cell death. **(E)** SB203580 pre-treatment for 1 h reduced Lp(a)-induced elevation of FerroOrange fluorescence intensity. **(F)** SB203580 pre-treatment for 1 h mitigated Lp(a)-induced increases in intracellular MDA levels. **(G)** p38 knockdown via siRNA inhibited Lp(a)-mediated upregulation of p-p38 and ACSL4 expression, as well as the downregulation of GPX4 expression. **(H)** p38 knockdown via siRNA prevented Lp(a)-induced cell death. **(I)** p38 knockdown via siRNA attenuated Lp(a)-induced enhancement of FerroOrange fluorescence intensity. **(J)** p38 knockdown via siRNA attenuated Lp(a)-induced increases in intracellular MDA levels. *P < 0.01 versus the control group; ^#^*P* < 0.01 versus the Lp(a) group. The values are expressed as mean ± SD (*n* = 6 per group).

Treatment of AC16 cardiomyocytes with Lp(a) induced p38 MAPK activation, as evidenced by a time-dependent increase in p-p38 levels, without significant changes in total p38 expression (Figure 2A). Compared with the control cells, confocal microscopy confirmed enhanced p-p38 fluorescence intensity in both the cytoplasmic and nuclear compartments of Lp(a)-treated cells (Figure 2B), indicating the spatial activation of p38 signaling. To assess the functional role of p38 in Lp(a)-mediated cytotoxicity, prior to exposure to Lp(a), cells were pre-treated with the p38 inhibitor SB203580 at the concentration of 10 μmoL/L, which markedly reduced LDH release (Figure 2D), suppressed intracellular ferrous iron accumulation (Figure 2E), attenuated lipid peroxidation (as indicated by MDA measurement) (Figure 2F), and inhibited the Lp(a)-induced upregulation of p-p38 and ACSL4, as well as the downregulation of GPX4 at the protein level (Figure 2C). This indicated that p38 activation exacerbates iron uptake and oxidative stress. Genetic validation via p38 siRNA knockdown similarly attenuated p-p38 expression, suppressed the Lp(a)-driven ferrous iron elevation and excessive MDA production, and reduced cardiomyocyte death (Figures 2H–J). Western blotting confirmed this suppression of ACSL4 and p-p38 expression in p38-silenced cells (Figure 2G). Collectively, the responses to these pharmacological and genetic interventions established p38 MAPK as a key mediator of Lp(a)-induced ferroptosis in cardiomyocytes, mediated via enhanced iron acquisition and lipid peroxidation cascades.

### Lp(a) activates p53 via p38-mediated signaling

**FIGURE 3:**
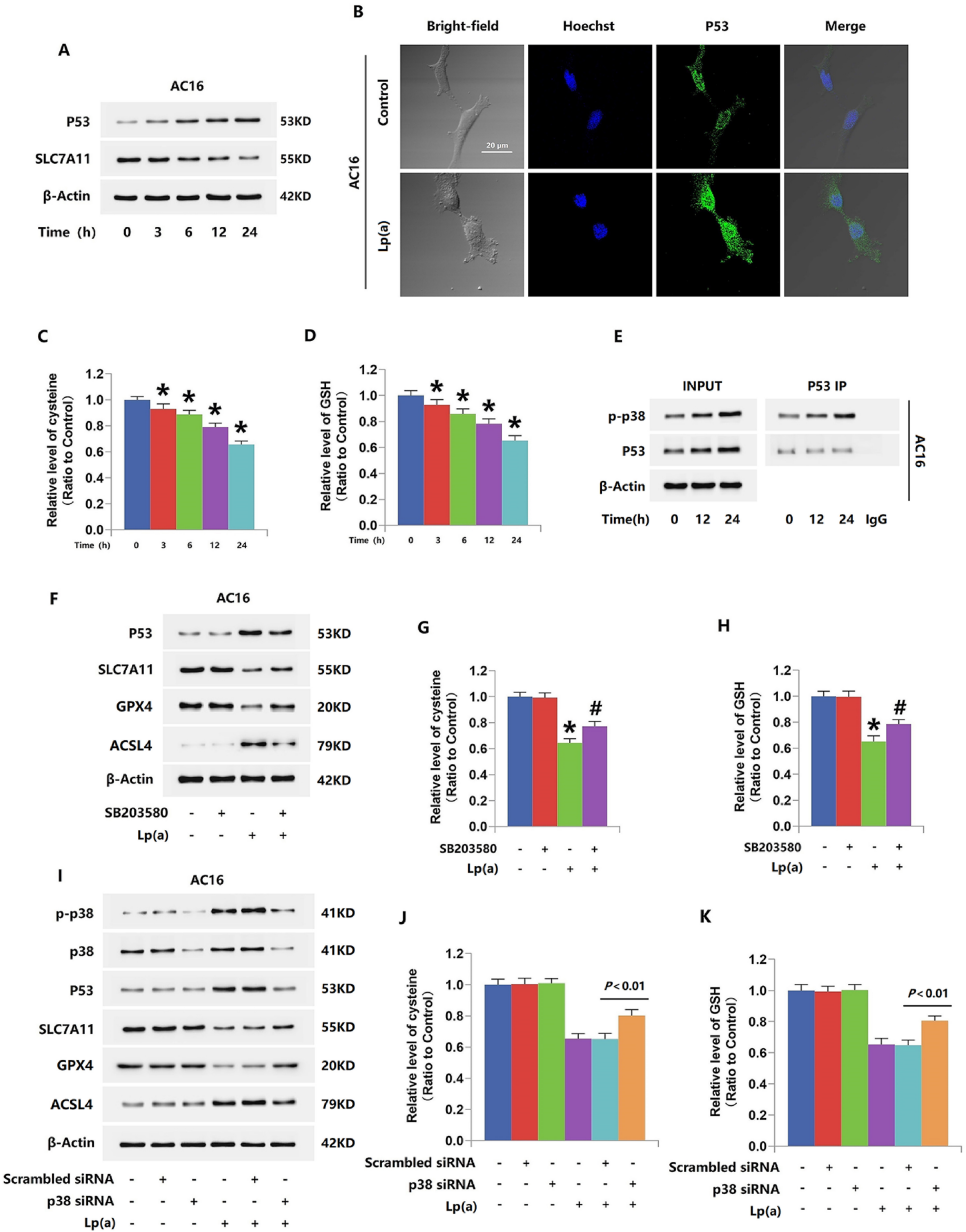
Lp(a) activates p53 through p38-mediated signaling. **(A)** Time-dependent upregulation of p53 protein expression and concurrent downregulation of SLC7A11 were observed in AC16 cells treated with 2 μmoL/L Lp(a) for 0, 3, 6, 12, and 24 h. **(B)** Confocal microscopy demonstrated increased p53 accumulation in both the cytoplasm and nuclei of AC16 cells following 24-h exposure to 2 μmoL/L Lp(a). **(C)** Intracellular cysteine levels exhibited a time-dependent decrease in AC16 cells treated with 2 μmoL/L Lp(a) across 0–24 h. **(D)** Intracellular glutathione levels showed a time-dependent reduction in AC16 cells treated with 2 μmol/L Lp(a) across 0–24 h. **(E)** Co-immunoprecipitation (Co-IP) combined with western blotting revealed that p-p38 co-precipitation with p53 increased progressively with prolonged Lp(a) exposure. **(F)** Pre-treatment with 10 μmoL/L SB203580 for 1 h attenuated Lp(a)-induced upregulation of p53 and ACSL4 as well as downregulation of SLC7A11 and GPX4. **(G)** SB203580 pre-treatment for 1 h mitigated Lp(a)-mediated cysteine depletion. **(H)** SB203580 pre-treatment for 1 h suppressed Lp(a)-induced glutathione reduction. **(I)** p38 knockdown via siRNA abolished Lp(a)-triggered p-p38 activation, upregulation of p53 and ACSL4 as well as downregulation of SLC7A11 and GPX4. **(J)** p38 knockdown via siRNA prevented Lp(a)-induced cysteine depletion. **(K)** p38 knockdown via siRNA inhibited Lp(a)-mediated GSH reduction. *P <0.01 versus the control group; #*P* <0.01 versus the Lp(a) group. The values are expressed as mean ± SD (*n* = 6 per group).

Exposure of AC16 cardiomyocytes to Lp(a) was observed to induce a time-dependent upregulation of p53 protein expression accompanied by a progressive downregulation of SLC7A11 (system xCT), as demonstrated by western blotting (Figure 3A). Compared with the control cells, confocal microscopy revealed an intensification of cytoplasmic and nuclear p53 fluorescence in treated cells (Figure 3B). Given the pivotal role of SLC7A11 in cystine/glutamate exchange for GSH biosynthesis, subsequent quantification confirmed time-dependent reductions in intracellular cysteine and GSH levels following Lp(a) treatment (Figures 3C,D), collectively indicating the Lp(a)-mediated disruption of redox homeostasis. Co-immunoprecipitation assays using anti-p53 antibodies revealed enhanced binding of p-p38 to p53 in Lp(a)-treated cells, with interaction intensities correlated with treatment duration (Figure 3E), indicating dynamic complex formation during ferroptosis progression. Moreover, pharmacological inhibition of p38 via 10 μmoL/L SB203580 pre-treatment suppressed Lp(a)-induced p53 and ACSL4 elevation, attenuated SLC7A11 and GPX4 downregulation, and partially restored cysteine and GSH depletion (Figures 3F–H). Similarly, genetic p38 silencing mediated via siRNA transfection abolished the Lp(a)-triggered upregulation of p-p38, p53, and ACSL4, mitigated the suppression of SLC7A11 and GPX4, and preserved cysteine and GSH levels (Figures 3I–K). These complementary interventions thus confirmed that p38-dependent p53 activation is a pivotal mechanism driving Lp(a)-induced cardiomyocyte ferroptosis, mediated by impaired cystine uptake, compromised antioxidant capacity, and dysregulated iron-redox cross-talk.

### p53 Mediates Lp(a)-induced cardiomyocyte ferroptosis

**FIGURE 4:**
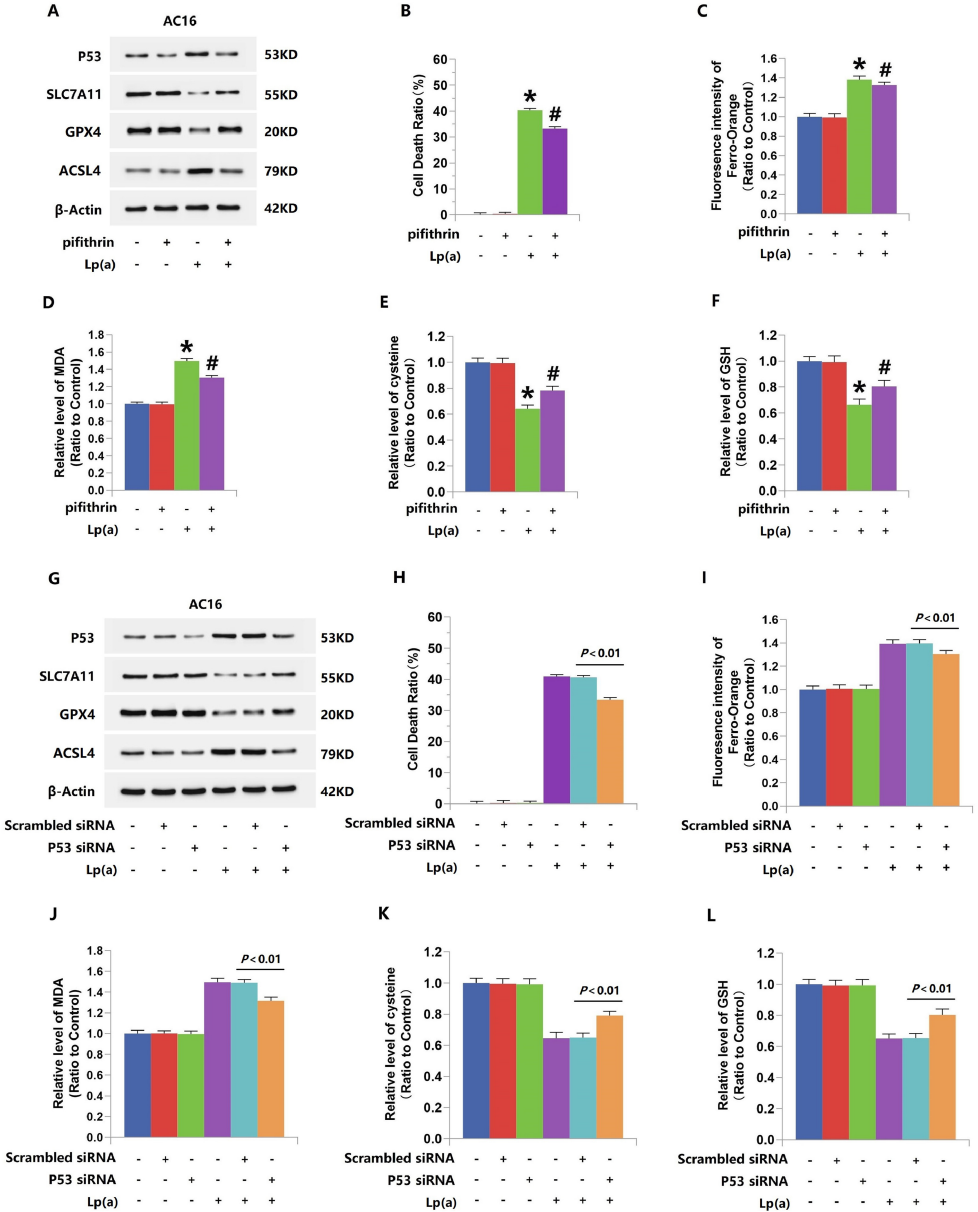
p53 mediates Lp(a)-induced cardiomyocyte ferroptosis. **(A)** Pre-treatment with 10 μmoL/L pifithrin-α for 1 h attenuated Lp(a)-induced upregulation of p53 and ACSL4 as well as downregulation of SLC7A11 and GPX4 in AC16 cells. **(B)** Pifithrin-α pre-treatment for 1 h suppressed Lp(a)-triggered cardiomyocyte death. **(C)** Pifithrin-α pre-treatment for 1 h mitigated Lp(a)-induced elevation of FerroOrange fluorescence intensity. **(D)** Pifithrin-α pre-treatment for 1 h reduced Lp(a)-elevated intracellular MDA levels. **(E)** Pifithrin-α pre-treatment for 1 h prevented Lp(a)-mediated cysteine depletion. **(F)** Pifithrin-α pre-treatment for 1 h inhibited Lp(a)-induced glutathione reduction. **(G)** p53 knockdown via siRNA abolished Lp(a)-triggered upregulation of p53 and ACSL4 as well as downregulation of SLC7A11 and GPX4. **(H)** p53 knockdown via siRNA attenuated Lp(a)-induced cardiomyocyte death. **(I)** p53 knockdown via siRNA suppressed Lp(a)-enhanced FerroOrange fluorescence intensity. **(J)** p53 knockdown via siRNA mitigated Lp(a)-elevated intracellular MDA levels. **(K)** p53 knockdown via siRNA reversed Lp(a)-mediated cysteine depletion. **(L)** p53 knockdown via siRNA prevented Lp(a)-induced glutathione reduction. *P < 0.01 versus the control group; #*P* < 0.01 versus the Lp(a) group. The values are expressed as mean ± SD (*n* = 6 per group).

To investigate the functional role of p53 in Lp(a)-mediated ferroptosis, AC16 cardiomyocytes were pre-treated with the p53 inhibitor pifithrin-α at a concentration of 10 μmol/L, which effectively suppressed Lp(a)-induced p53 upregulation, mitigated ACSL4 overexpression, and attenuated downregulation of SLC7A11 and GPX4 (Figure 4A). The findings of corresponding LDH release assays revealed the protective effects of pifithrin-α against Lp(a)-triggered cell death, whereas intracellular analyses revealed its inhibition of ferrous iron accumulation, lipid peroxidation (as measured by MDA quantification) (Figures 4B–D), and partial restoration of cysteine and GSH (Figures 4E,F), thus confirming the regulatory involvement of p53 in ferroptotic cascades. Genetic validation via p53 siRNA knockdown corroborated these findings, revealing alleviated suppression of SLC7A11 and GPX4, blunted elevations in ferrous iron and MDA, maintained cysteine and GSH levels, and diminished upregulation of ACSL4 in Lp(a)-treated cells (Figures 4G–L). Collectively, these pharmacological and genetic interventions indicated that Lp(a) activates the p38–p53 axis, thereby impairing cystine/GSH metabolism by suppressing SLC7A11, and promoting lipid peroxidation-dependent ferroptosis in cardiomyocytes, thus identifying p53 as a central mediator in this pathological cascade.

### ROS contributes to the Lp(a)-dependent p38 activation

**FIGURE 5:**
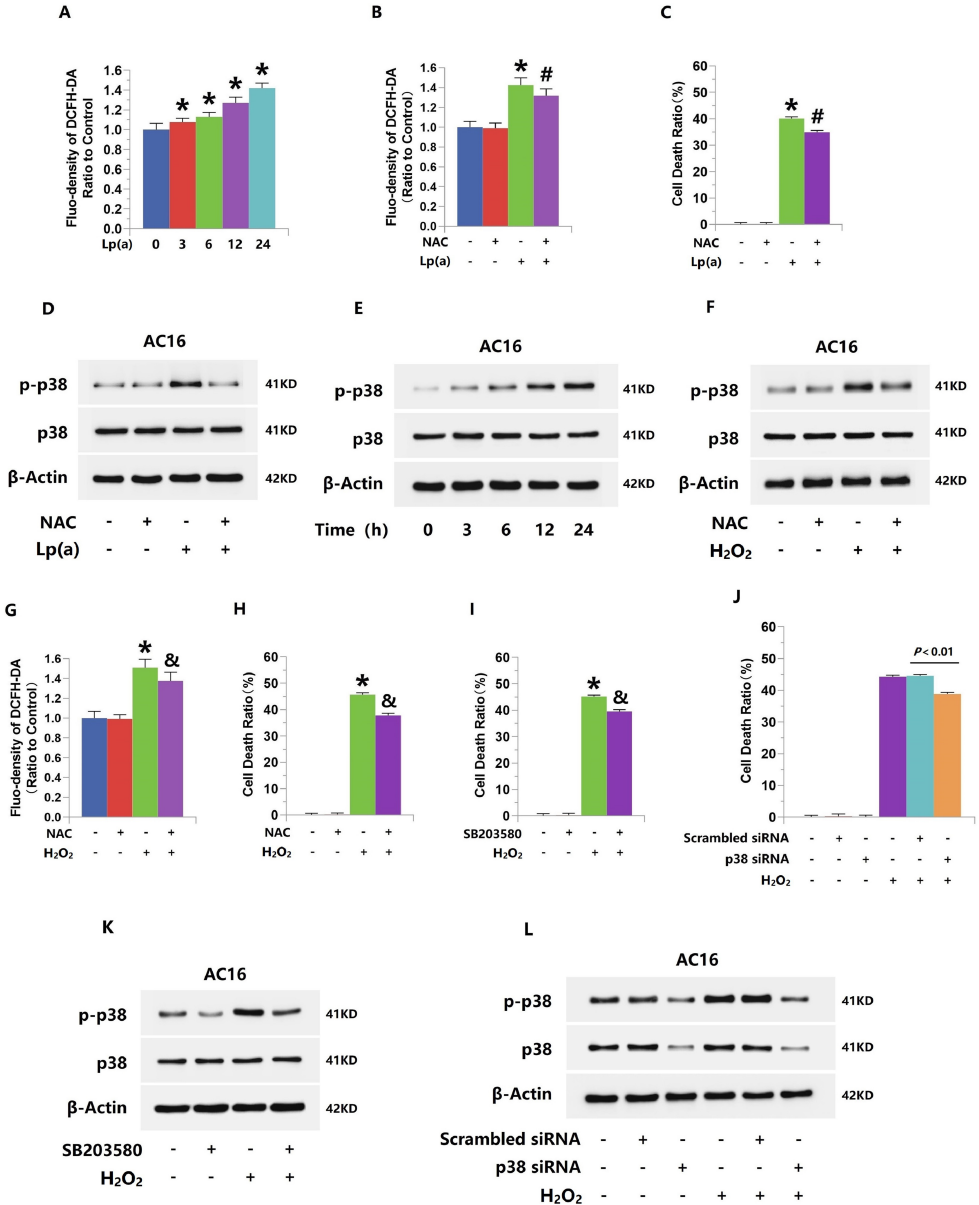
ROS contributes to Lp(a)-dependent activation of p38. **(A)** AC16 cells treated with 2 μmoL/L Lp(a) for 0–24 h exhibited a time-dependent increase in intracellular ROS levels. **(B)** Pre-treatment with 10 mmoL/L N-acetylcysteine (NAC, 1 h) suppressed Lp(a)-induced ROS elevation. **(C)** NAC pre-treatment for 1 h attenuated Lp(a)-triggered cell death. **(D)** NAC pre-treatment for 1 h inhibited Lp(a)-induced upregulation of phosphorylated p38 (p-p38). **(E)** AC16 cells exposed to 50 μmoL/L H_2_O_2_ for 0–24 h showed time-dependent p-p38 upregulation. **(F)** NAC pre-treatment for 1 h abolished H_2_O_2_-induced p38 activation. **(G)** NAC pre-treatment for 1 h mitigated H_2_O_2_-driven ROS accumulation. **(H)** NAC pre-treatment for 1 h prevented H_2_O_2_-induced cell death. **(I)** SB203580 pre-treatment for 1 h (10 μmoL/L) suppressed H_2_O_2_-mediated cell death. **(J)** p38 knockdown via siRNA abolished H_2_O_2_-triggered cell death. **(K)** SB203580 pre-treatment for 1 h inhibited H_2_O_2_-induced p38 phosphorylation. **(L)** p38 knockdown via siRNA prevented H_2_O_2_-driven p-p38 upregulation. *P < 0.01 versus the control group; #*P* < 0.01 versus the Lp(a) group; &*P* < 0.01 versus the H_2_O_2_ group. The values are expressed as mean ± SD (*n* = 6 per group).

With respect to elucidating the mechanistic basis of p38 activation in Lp(a)-treated cardiomyocytes, AC16 cells exposed to 2 μmoL/L Lp(a) showed a time-dependent accumulation of intracellular ROS from 0 to 24 h (Figure 5A), whereas pre-treatment with the ROS scavenger NAC at the concentration of 10 mmoL/L for 1 h effectively attenuated Lp(a)-induced elevation in ROS levels (Figure 5B) and concurrent cell death (Figure 5C). These findings suggested that ROS were key mediators of Lp(a) cytotoxicity. Western blotting revealed that NAC suppressed the Lp(a)-triggered p-p38 upregulation (Figure 5D), thereby directly linking the excessive ROS production with p38 activation. Moreover, exogenously administered hydrogen peroxide (H_2_O_2_) promoted a pronounced increase in p-p38 expression, which was abolished by NAC pre-treatment (Figures 5E,F), whereas NAC also mitigated H_2_O_2_-driven ROS accumulation and cell death (Figures 5G,H), confirming the functionality of the ROS–p38 signaling axis. Pharmacological inhibition of p38 induced by pre-treatment with SB203580 not only blocked H_2_O_2_-induced p38 activation but also reduced cell mortality, a finding corroborated by p38 siRNA silencing experiments, which revealed attenuated p-p38 expression and enhanced cell survival (Figures 5I–L). These sequential interventions, including ROS modulation, pharmacological inhibition, and genetic silencing, conclusively demonstrated that Lp(a)-induced cardiomyocyte death occurs via ROS-dependent p38 activation, with p38 serving as both a redox-sensitive kinase and central executor of oxidative cytotoxicity.

### p38/p53 Signaling mediates Lp(a)-induced ferroptosis *in vivo*

**FIGURE 6:**
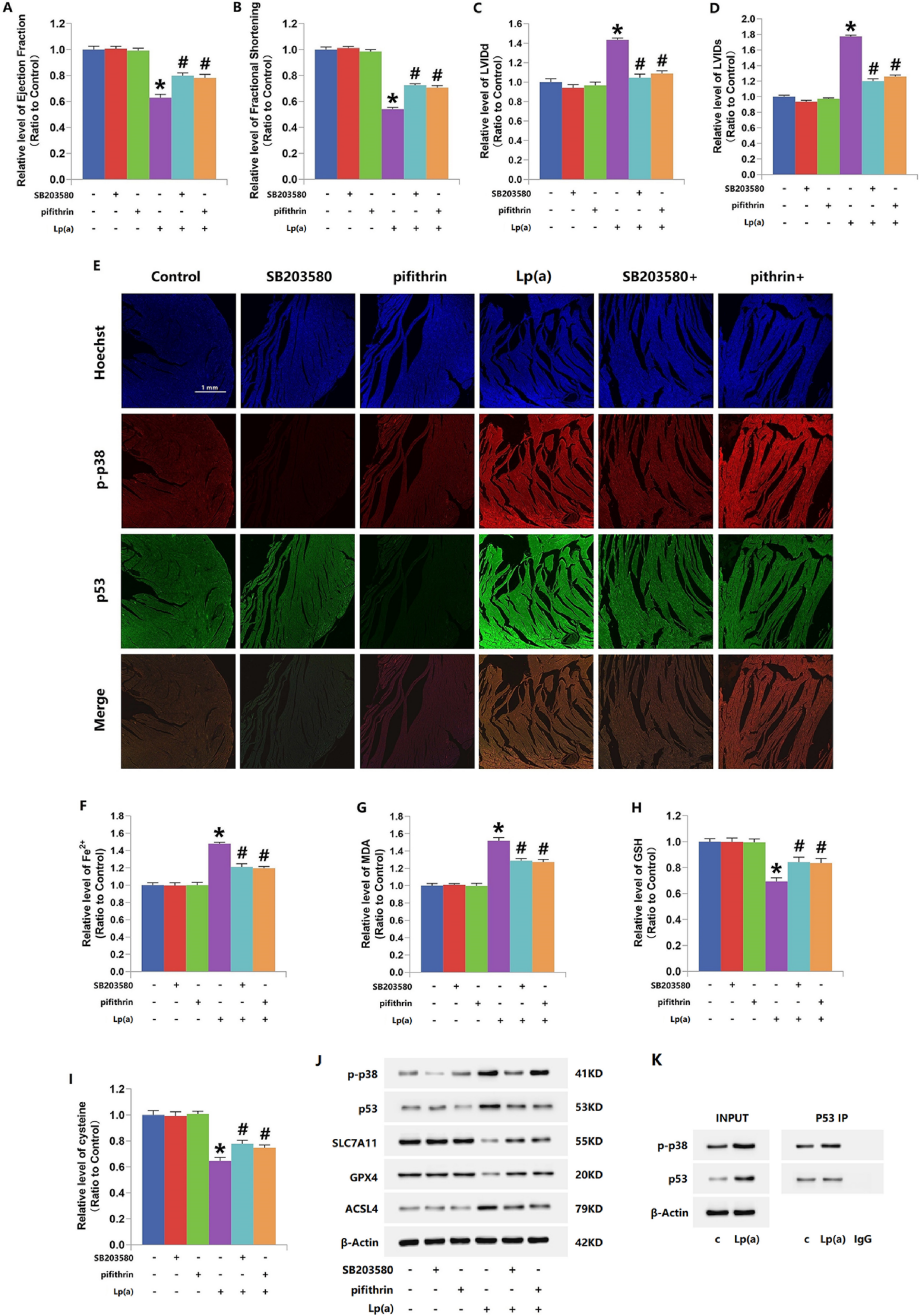
p38/p53 signaling mediates Lp(a)-induced ferroptosis *in vivo*. **(A,B)** Pre-treatment with SB203580 (5 mg/kg) or pifithrin-α (2.2 mg/kg) attenuated Lp(a)-induced reductions in left ventricular ejection fraction (EF) and fractional shortening (FS) in mice. **(C,D)** SB203580/pifithrin-α pre-treatment suppressed Lp(a)-elevated left ventricular internal diastolic dimension (LVIDd) and left ventricular internal dimension systole (LVIDs). **(E)** Confocal microscopy demonstrated that Lp(a) upregulated myocardial p-p38 and p53 expression, SB203580 pre-treatment inhibited Lp(a)-induced p-p38/p53 co-activation, and pifithrin-α pre-treatment specifically blocked p53 induction. **(F,G)** SB203580/pifithrin-α pre-treatment mitigated Lp(a)-elevated ferrous iron and MDA levels. **(H,I)** SB203580/pifithrin-α pre-treatment prevented Lp(a)-induced glutathione depletion and cysteine deficiency. **(J)** SB203580 abrogated the lipoprotein(a)-induced upregulation of p-p38, p53, and ACSL4, as well as the downregulation of SLC7A11 and GPX4; pifithrin-α inhibited the lipoprotein(a)-triggered upregulation of p53 and ACSL4 and the concomitant downregulation of SLC7A11 and GPX4. **(K)** Co-immunoprecipitation assays revealed that lipoprotein(a) promotes the binding between p-p38 and p53. *P < 0.01 versus the control group; #*P* < 0.01 versus the Lp(a) group. The values are expressed as mean ± SD (*n* = 6 per group).

Echocardiographic assessment after 3 days of daily tail vein administration revealed a significant impairment of the ejection fraction in the Lp(a)-treated mice compared with that in control mice (Figures 6A–D). Subsequent histopathological analysis of paraffin-embedded cardiac sections via immunofluorescence revealed marked elevations in p-p38 and p53 expression in Lp(a)-exposed myocardium, with SB203580 pre-treatment reducing both p-p38 and p53 levels. Meanwhile, pifithrin-α specifically attenuated p53 expression compared with that in mice treated only with Lp(a) (Figures 6E,J), indicating sequential p38-dependent p53 activation during the progression of cardiac injury. Furthermore, biochemical quantification of cardiac tissues revealed Lp(a)-induced increases in ferrous iron and MDA production, with corresponding depletion of GSH and cysteine. These ferroptotic markers were substantially mitigated in both the SB203580 and pifithrin-α pre-treated groups (Figures 6F–I). Western blot results demonstrated that lipoprotein(a) upregulated the expression of p-p38, p53, and ACSL4 while downregulating SLC7A11 and GPX4 *in vivo*. SB203580 abrogated the lipoprotein(a)-induced upregulation of p-p38, p53, and ACSL4, as well as the downregulation of SLC7A11 and GPX4; pifithrin-α inhibited the lipoprotein(a)-triggered upregulation of p53 and ACSL4 and the concomitant downregulation of SLC7A11 and GPX4 (Figure 6J). Co-immunoprecipitation assays revealed that lipoprotein(a) promotes the binding between p-p38 and p53 (Figure 6K). These coordinated functional, molecular, and biochemical findings confirmed that Lp(a) induces cardiomyocyte ferroptosis *in vivo* by activating the p38–p53 axis, driving iron dysregulation and lipid peroxidation while impairing antioxidant defense mechanisms.

## Discussion

This study, for the first time, demonstrated that Lp(a) can trigger ferroptosis in cardiomyocytes. Ferroptosis is an iron-dependent form of cell death, initiated by abnormally elevated intracellular ferrous ions resulting from disrupted iron homeostasis of the movement of iron ions in and out of the cell (19), while it has also been linked to several cardiomyopathies of distinct etiologies (20). TF and TFR1-mediated endocytosis translocate iron into the cell, whereas ferroportin in the plasma membrane expels iron from cells. Following TFR upregulation, an increase in intracellular iron levels leads to ferroptosis (21), whereas downregulation of TFR1 has the opposite effect, preventing ferroptosis (22). In the present study, AC16 cell death occurred in response to the addition of 2 μmoL/L Lp(a) in a time-dependent manner. In addition, compared with that in the control group, we detected an increase in the intracellular ferrous ion levels following Lp(a) addition, whereas pre-treatment of cells with FAC promoted cell death, and pre-treatment with FER-1 and DFO inhibited cell death. Furthermore, prolonged Lp(a) exposure altered the intracellular levels of TF, TFR1, and FPN proteins, indicating that Lp(a) modulates iron transfer to alter intracellular iron ion concentrations by regulating TF and FPN expression. Concurrently, we also detected a decline in the expression of SLC7A11, cysteine, and GSH, whereas following Lp(a) addition, lipid peroxidation increased. These findings confirmed that Lp(a) promotes ferroptosis occurrence in AC16 cells. Simultaneously, we detected p38 and p53 activation, whereas the levels of ferroptosis in cardiomyocytes were suppressed following the inhibition of p38 activity using SB203580 or p38 siRNA. These findings suggested that the Lp(a)-induced ferroptosis in cardiomyocytes is mediated by p38 activation. In addition, p38 promoted the Lp(a)-induced upregulated expression and nuclear translocation of p53 in cardiomyocytes, which further inhibited SLC7A11 activity, reduced intracellular levels of cysteine and GSH, and promoted cardiomyocyte ferroptosis. Ferroptosis in cardiomyocytes was also suppressed by the inhibition of p53 activity using pifithrin-α and a p53 siRNA. This indicates that Lp(a)-induced ferroptosis in cardiomyocytes is mediated by p38 activation, which in turn activates p53. Finally, elevated ROS levels in cardiomyocytes were associated with p38 activation, and Lp(a) could promote increases in ROS levels in cardiomyocytes. This implies that Lp(a) activates p38 in these cells by elevating ROS levels. Collectively, these findings indicate that Lp(a) induces cardiomyocyte ferroptosis via the p38–p53 signaling axis.

Lp(a) was first identified by Norwegian geneticists in studies focusing on LDL (23). Its concentration in cells is mainly genetically determined and varies considerably among different populations. Lp(a) has now been established as a residual risk factor for cardiovascular disease and a frequent research focus due to its important role in cardiovascular disease and contribution to coronary atherosclerosis, which is also closely associated with heart failure. The Lp(a)-associated risks of heart failure and heart failure with preserved ejection fraction have previously been identified as prominent factors in participants of a multi-ethnic study (24). A positive correlation between Lp(a) and heart failure has also been confirmed based on systematic evaluation (25). Notably, previous studies have indicated an association between Lp(a) and myocardial damage. Similarly, we also observed a time-dependent increase in the mortality of AC16 cardiomyocytes following Lp(a) treatment, whereas treatment with DFO and FER-1 significantly inhibited damage caused by Lp(a). Further analysis of the intracellular levels of ferrous ions, lipid peroxidation levels, and changes in the expression of iron transporter proteins indicated that Lp(a) triggered ferroptosis in cardiomyocytes.

The MAPK signaling cascade comprises three-tiered kinase modules—MAPK kinase kinases, MAPK kinases, and terminal MAPKs—that are sequentially activated to regulate vital cellular processes, including proliferation, differentiation, stress adaptation, and inflammatory responses. Among its four principal branches (ERK, JNK, p38, and ERK5), p38, a 38-kDa stress-responsive kinase in the p38 MAPK pathway, is rapidly phosphorylated at tyrosine residues in response to LPS challenge (26, 27) and serves as a signaling nexus governing inflammation, apoptosis, and tumour genesis through an intricate pattern of cross-talk with parallel pathways. p38 is a ferroptosis modulator, and in acute lung injury, IL-17A-induced alveolar epithelial ferroptosis requires ACT1/TRAF6/p38 signaling (28). Meanwhile, endometrial stromal cells involve IL-33-activated p38/JNK/ATF3 pathways to upregulate SLC7A11 and suppress ferroptosis (29). Notably, ionizing radiation exacerbates atherosclerosis via p38/NCOA4-mediated endothelial ferroptosis (30). In the present study, we established that Lp(a)-treated AC16 cardiomyocytes are characterized by elevated p-p38 levels, concomitant with SLC7A11 suppression, cysteine depletion, and GSH exhaustion, which are hallmarks of iron-dependent death abrogated by p38 inhibition (by SB203580 or p38 siRNA). This mechanistic convergence confirms the central role of p38 in orchestrating cardiomyocyte ferroptosis through redox-sensitive transcriptional regulation, thus broadening its pathophysiological repertoire beyond classical inflammatory paradigms.

Previous research has established that p38 MAPK can activate p53 via phosphorylation at serine residues 15 and 46 (the key post-translational modification sites), regulated by multiple protein kinases, including MAPK family members (31, 32). However, these regulatory mechanisms are often characterized by cell type-specific and stimulus-dependent variations (33–35). The p53 tumor suppressor gene, located on human chromosome 17p13.1, encodes the 53-kDa nuclear phosphoprotein that functions as a key regulator of cell cycle progression, DNA repair, differentiation, and apoptosis. Although previous studies have focused predominantly on p38-mediated p53 activation in apoptosis and autophagic cell death, limited research has explored its potential involvement in alternative cell death processes. The ability of p53 to promote ferroptosis through distinct mechanisms has been linked to the upregulation of glutaminase 2, which enhances glutamine catabolism to α-ketoglutarate, thereby amplifying mitochondrial respiratory chain activity and ROS accumulation (22). Similarly, p53 promotes ferroptosis by activating SAT1, accelerating polyamine metabolism and ROS generation, ultimately exacerbating lipid peroxidation (36). Moreover, p53 modulates ferroptosis directly by repressing SLC7A11 (xCT), the catalytic subunit of a cystine/glutamate antiporter essential for cystine uptake and subsequent GSH biosynthesis, which is the main antioxidant defense against ferroptosis. Mechanistically, p53 binds to the SLC7A11 promoter, inhibiting its transcription, reducing cystine availability, limiting GSH synthesis, and compromising cellular antioxidant capacity, thus contributing to elevated lipid peroxidation-mediated ferroptosis (37). Our findings revealed that Lp(a)-induced p38 activation in cardiomyocytes triggers the sequential activation of p53, leading to SLC7A11 suppression and subsequent ferroptosis. This suggests that, pathophysiologically, the roles of p38–p53 signaling may extend beyond the classical apoptotic pathways.

ROS, a group of oxygen-derived molecules, including superoxide anions, H_2_O_2_, and hydroxyl radicals (OH), function as key redox regulators under physiological conditions to maintain redox homeostasis, modulating essential signaling pathways involved in inflammation, proliferation, apoptosis, and adaptation to hypoxia (38, 39). However, under redox imbalance, ROS can act as pathogenic mediators that promote inflammatory and oncogenic processes (40), with ferroptosis being particularly dependent on ROS-mediated lipid peroxidation and subsequent mitochondrial structural alterations (41). Mechanistically, ROS activates p38 MAPK via multiple pathways, including the oxidation of apoptosis signal-regulating kinase 1, suppression of phosphatase activity, and activation of the Rac1/NADPH oxidase complex, to orchestrate cellular responses to oxidative stress and inflammatory signaling (42). Under pathological conditions, excessive ROS induce apoptosis or necrosis by activating the MAPK and caspase cascades (43–46), as demonstrated in various cellular backgrounds, including fibroblasts (47), neural stem cells (48), U937 lymphocytes (49), and A549 pulmonary epithelial carcinoma cells (50). The present study revealed that pre-treatment with NAC, an intracellular ROS scavenger, effectively suppressed Lp(a)-induced p38 activation, whereas exogenously administered H_2_O_2_ directly stimulated p38 phosphorylation. These findings enabled the identification of a definitive ROS–p38 axis that subsequently activates p53 to induce ferroptosis, thus providing mechanistic insights into redox-regulated cell death pathways.

Despite these important findings, we limited our investigation to assessing the effects of Lp(a) on myocardial cells *in vitro* and in murine models *in vivo*, which may not fully recapitulate the human physiological environment.

In conclusion, this study revealed a novel redox-sensitive mechanism in which an Lp(a)-induced accumulation of ROS triggers p38 activation, which subsequently induces the nuclear translocation of p53 to transcriptionally repress SLC7A11, depleting GSH reserves and inducing ferroptosis in human cardiomyocytes. Importantly, we identified Lp(a) as the first reported inducer of cardiomyocyte ferroptosis and also delineated the p38–p53 axis as a master regulatory pathway for iron-dependent cell death. These findings contribute to enhancing our understanding of non-apoptotic signaling in cardiovascular pathophysiology. In the future, we plan to secure regulatory approval for clinical studies to further elucidate the mechanisms underlying Lp(a) action on human cardiomyocytes to identify new targets and directions for future treatment of myocardial injury.

## Data Availability

The original contributions presented in the study are included in the article/supplementary material, further inquiries can be directed to the corresponding author.
